# The future of the London Buy-To-Let property market: Simulation with temporal Bayesian Networks

**DOI:** 10.1371/journal.pone.0179297

**Published:** 2017-06-27

**Authors:** Anthony C. Constantinou, Norman Fenton

**Affiliations:** Risk and Information Management (RIM) Research Group, School of Electronic Engineering and Computer Science, Queen Mary University of London, London, United Kingdom; Tongji University, CHINA

## Abstract

In 2015 the British government announced a number of major tax reforms for individual landlords. To give landlords time to adjust, some of these tax measures are being introduced gradually from April 2017, with full effect in tax year 2020/21. The changes in taxation have received much media attention since there has been widespread belief that the new measures were sufficiently skewed against landlords that they could signal the end of the Buy-To-Let (BTL) investment era in the UK. This paper assesses the prospective performance of BTL investments in London from the investor’s perspective, and examines the impact of incoming tax reforms using a novel Temporal Bayesian Network model. The model captures uncertainties of interest by simulating the impact of changing circumstances and the interventions available to an investor at various time-steps of a BTL investment portfolio. The simulation results suggest that the new tax reforms are likely to have a detrimental effect on net profits from rental income, and this hits risk-seeking investors who favour leverage much harder than risk-averse investors who do not seek to expand their property portfolio. The impact on net profits also poses substantial risks for lossmaking returns excluding capital gains, especially in the case of rising interest rates. While this makes it less desirable or even non-viable for some to continue being a landlord, based on the current status of all factors taken into consideration for simulation, investment prospects are still likely to remain good within a reasonable range of interest rate and capital growth rate variations. The results also suggest that the recent trend of property prices in London increasing faster than rents will not continue for much longer; either capital growth rates will have to decrease, rental growth rates will have to increase, or we shall observe a combination of the two events.

## Introduction

In 1998 the *Assured shorthold tenancy*—a form of assured tenancy with limited security—was introduced in Britain. This type of tenancy constituted a major shift in favourability towards landlords, leading to an explosion of interest in Buy-To-Let (BTL) property investments in the UK from both amateur and savvy investors, at both national and international level. Even amateur investors have been able to develop enormous portfolios. The case of the Wilsons’ is instructive [1]; this couple of ex-maths teachers had started buying homes to let in the late 1980s. In 2015, they were reported to be Britain’s biggest BTL couple, at which point they started selling off their property empire, which consisted of approximately 1,000 properties, with a target to make £100m profit.

Success stories like these demonstrate the maximum (observed) potential of a BTL investment. Such vast profits can be made with increased amounts of borrowed capital, known as *leverage*, under the expectation that profits made will be greater than the interest paid on the additional borrowed capital. The high levels of lending to landlords was the basis for the Bank of England to express concerns on numerous occasions about the UK’s property market. The problem with excessive amounts of leveraging is that, in the event of a crisis, many investors with excessive amounts of borrowing may be forced to sell at the same time, and this poses a threat to the UK’s financial stability. This is, at least, part of the reason why the UK government has announced significant tax reforms specifically to target BTL investors.

There have been arguments in the media that, as an indirect effect of these measures, investors themselves may seek to offset part of their prospective losses by increasing rents, potentially affecting the lives of Londoners who already struggle to find affordable accommodation. However, these tax measures solely focus on BTL investors and hence, it is vital that we first understand the resulting direct effects on investors prior to assessing how the tax changes may indirectly influence the aggregate market. As a result, this paper introduces a novel temporal Bayesian Network (BN) simulator to assess the prospective impact of the various tax changes from the perspective of BTL investors, by taking into account a range of factors and uncertainties.

The paper is structured as follows: The new tax measures are described in detail in Section 2. Section 3 describes the factors being simulated. We provide a brief overview of BNs in Section 4 before describing the temporal BN model employed for simulation in Section 5. The simulation results are presented in Section 6 and discussed in Section 7. Finally, we provide our concluding remarks in Section 8.

## New tax measures for landlords

This section summarises the changes in taxation for landlords. The new measures involve three key points: a) interest related tax relief, b) stamp duty land tax, and c) wear and tear allowance.

### Interest related tax relief

Under the existing system, all property expenses can be deducted from income when computing tax to be paid. Currently, such expenses receive tax relief at the landlord’s highest income tax rate (e.g. 40% when in the *Higher rate* tax band; refer to Table 1). Under the new system, interest payments will be treated differently to other expenses. Specifically, they will be capped for tax relief at the *Basic rate* of 20% [2].

**Table 1:** Current income tax rates and bands in the UK [3].

Band	Taxable income	Tax rate
Personal Allowance	Up to £11,000	0%
Basic rate	£11,001 to £43,000	20%
Higher rate	£43,001 to £150,000	40%
Additional rate	Over £150,000	45%

Since this new measure is restricted to the *Basic rate* income tax band, it gives the impression that those whose income does not surpass the *Basic rate* income tax band will remain unaffected. However, since interest is not offset against income, everyone’s gross profits increase under the new system and hence, this will inevitably push a number of *Basic rate* tax payers into the *Higher rate* band that will require them to pay more tax. The new measures seem to move towards taxing turnover rather than actual profits. Note that this measure does not apply to limited companies as they are subject to corporate tax.

To give landlords time to adjust, the government is introducing this change gradually from April 2017, and over four years, with the new measures fully implemented in tax year 2020/21. Table 2 shows how the new measures will affect net profit over this adjustment period, based on a hypothetical example.

**Table 2:** Illustration of the new measures through the adjustment period, based on a hypothetical example.

	Today 2016/17	Adjustment period			New measures 2020/21
		2017/18	2018/19	2019/20	
*Rental income*	£20,000	£20,000	£20,000	£20,000	£20,000
*Expenses (excluding interest related)*	£7,000	£7,000	£7,000	£7,000	£7,000
*Interest related expenses*	£8,000	£8,000	£8,000	£8,000	£8,000
*Tax relief from interest related expenses*	100% (£8,000)	75% (£6,000)	50% (£4,000)	25% (£2,000)	0% (£0)
*Gross profit*	£5,000	£7,000	£9,000	£11,000	£13,000
*Tax on gross profit*	£2,000	£2,800	£3,600	£4,400	£5,200
*Tax credit*	£0	£400	£800	£1,200	£1,600
*Tax bill*	£2,000	£2,400	£2,800	£3,200	£3,600
***Net profit***	£3,000	£2,600	£2,200	£1,800	£1,400

### Stamp duty land tax

The Stamp Duty Land Tax (SDLT) is a tax on land transactions in the UK, excluding Scotland where the *Land and Buildings Transaction Tax* was introduced in April 1, 2015, to replace SDLT [4]. In 2015 the UK government announced higher rates of SDLT on purchases of additional residential properties [5], effective from April 2016.

On the whole, the changes in SDLT increase the tax rate on additional residential properties by 3%, in absolute terms, in each band (refer to Table 3). The overall relative effect, however, is significant. For example, if we had bought an additional property for £300,000, prior to April 2016, the SDLT would have been £5,000, whereas today this tax figure is £14,000. Note that transactions under £40,000 are not subject to the higher rates. Table 4 illustrates how the additional tax is accumulated based on this example.

**Table 3:** SDLT changes in the UK for additional residential properties, effective from April 2016 [5].

Band	Old SDLT rates	New SDLT rates
£0–£125,000	0%	3%
£125,001–£250,000	2%	5%
£250,001–£925,000	5%	8%
£925,001–£1,500,000	10%	13%
Over £1,500,000	12%	15%

**Table 4:** The increase in the SDLT tax bill with the new measures, based on buying a property at £300,000.

Band	Tax based on the previous SDLT rates	Tax based on the new SDLT rates
£0–£125,000	£0	£3,750
£125,001–£250,000	£2,500	£6,250
£250,001–£925,000	£2,500	£4,000
**Total**	£5,000	£14,000

Note that the above changes in SDLT are in addition to those effective from December 2014, where the UK government wanted to make tax payments fairer by cutting tax for 98% of the people buying a home for less than £937,500, and increasing tax for the 2% of people who buy a property above that threshold [6]. The SDLT changes of 2014 applied to all residential properties.

### Wear and tear allowance

For furnished residential properties, the wear and tear allowance allowed landlords to deduct 10% of the gross rental income from gross profits. This allowance was supposed to represent the average costs of buying or replacing furniture or other capital items in the let property. The new measures, effective from April 1 2016, replaced this allowance with a relief that requires landlords to deduct the costs they actually incur on replacing (not servicing) furnishings, appliances and kitchenware in the property [7].

While this new measure may not appear to be as important as those described in Sections 2.1 and 2.2, it is expected to have a noticeable impact for two reasons. First, it further increases tax bills on the basis that the previous 10% was largely considered generous (hence the change). Second, this new measure requires additional effort from landlords to keep track of costs and receipts, which becomes tedious for those who own multiple properties.

## Factors being simulated

Before we describe the model and discuss the results from simulation, we first give a brief introduction to the key factors being simulated.

### Capital growth

Official price-paid data, for residential properties in London, is available online with open access by Land Registry [8]. However, these data are limited to 1995 onwards. Older price-paid data is retrieved by Nationwide, who are the largest building society in the UK as well as in the world [9].

**Fig 1:**
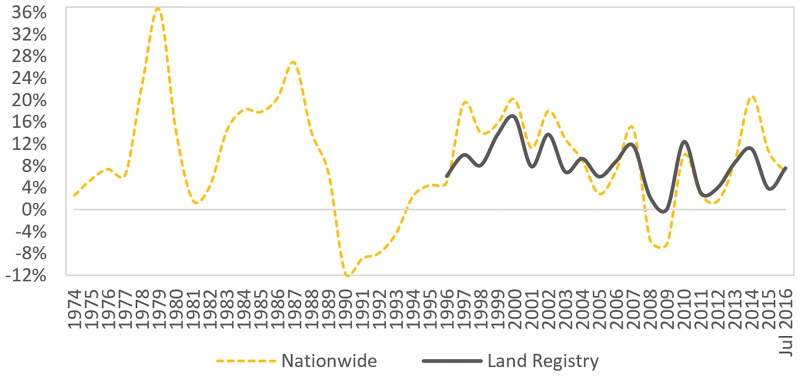
Annual capital growth for residential properties in London.

Fig 1 illustrates the annual capital growth generated by Nationwide data for years 1974 to June 2016, and Land Registry data for years 1996 to July 2016. The variation in capital growth between the two dataset is the result of Nationwide data capturing only part of the property sales in London, whereas Land Registry captures all of them. Land Registry data indicates that the average London property has experienced an average annual capital growth of 8.13% from 1995 to July 2016. On the other hand, Nationwide indicates that the growth for this same period has been 9.24% (though up to June, instead of July, 2016), and 8.7% from 1973 (4th quarter) to June 2016.

Fig 1 also captures the three major property market crises. The first was in the early 1980s when a severe global economic recession affected much of the develop world. The second occurred in 1992, also known as *Black Wednesday* [10], when the British government raised interest rates to unstable levels in an effort to control the rate of GBP to meet the limits introduced by the European Exchange Rate Mechanism (ERM). Eventually, the government was unable to keep the GBP above its agreed lower limit and was forced to withdraw from the ERM, which resulted in a fall in interest rates (refer to Section 3.3) and an end to the falling house prices (refer to Fig 1).

The third housing crisis came as a result of the 2007–09 *Global financial crisis*. The cause of this crisis was the burst of the USA housing ‘bubble’ due to a high default rate in the subprime home mortgage sector, as a result of many high-risk loans sold on as low-risk securities [11]. The crisis threatened the collapse of large financial institutions, with Lehman Brothers, the fourth largest investment bank in the USA, declaring bankruptcy in 2008. The crisis had an immediate negative effect on the London property market, though considerably lesser than the *Black Wednesday* crisis, as shown in Fig 1.

### Rental yield and rental growth

Private rental market statistics for London are available from the Valuation Office Agency [12], though the data used to generate these statistics is stated to be based on a sample of rental information. Their statistics indicate that the average rent in London was £1,727 between 1 April 2015 and 31 March 2016, based on a sample size of 62,810. Linking this to the Land Registry [8] data generates an average rental yield of approximately 3.7%.

We have also examined additional rental information which is publicly available by LendInvest [13], who are the world’s largest peer-to-peer platform for property. LendInvest statistics show that the rental yields in London spread between 4.5% and 7.4%. However, these yields are based on rental prices extracted from Zoopla for the period January 1 2015 to February 18 2016, and are calculated relative to Land Registry 2010 price-paid data. These statistics are overrated for two reasons: a) rental prices are compared to property values dated five to six years back, and b) rental prices on Zoopla represent market advertised rents and not real rents from tenancy agreements.

Portico estate agency’s interactive rental yield map indicates that rental yields in London spread between 2.2% and 5.5% [14], which are in agreement with the statistics retrieved by the Valuation Office Agency. Portico statistics show that in 2012 half of London was generating rental yields in excess of 6%, whereas since 2015 all London districts are generating rental yields below 6%. This implies that property price growth in London has been increasing faster than rents, at least over the last few years, hence pushing rental yields down. Portico state their statistics are updated daily based on several hundred estate agents in London, and reflect the three most recent months.

**Fig 2:**
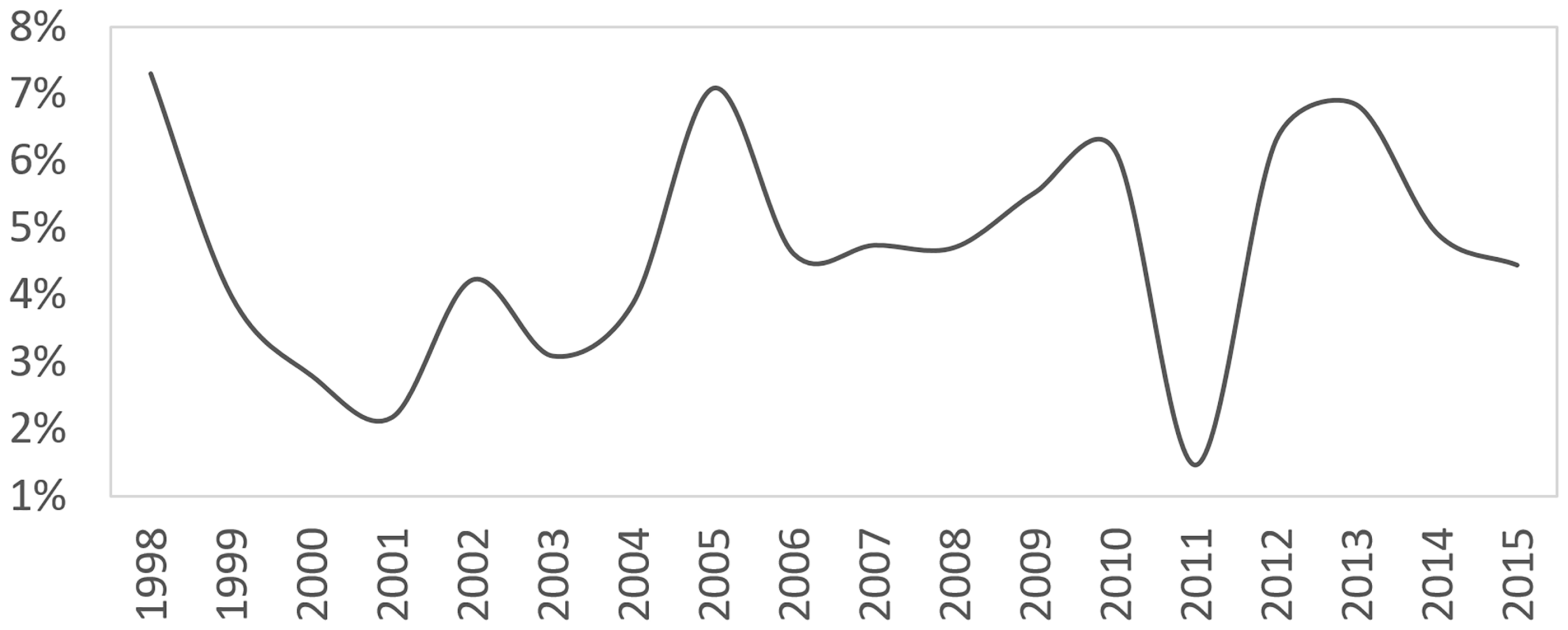
Annual rental income growth for local authority properties in London.

In the case of rental growth, we found official local authority rental data from the Department for Communities and Local Government [15]. We were unable to find relevant private rental income data. Fig 2 illustrates the annual rental income growth since 1998, which averages at 4.69%. Interestingly, local authority rents have been growing approximately half as fast as property prices, which inevitably decreases rental yields over each subsequent year, and which is in agreement with Portico’s [14] results. While local authority rents differ from private rents, we expect the two to correlate well.

However, it is reasonable to assume that the growth in the private sector has been slightly higher than the growth in the local authority sector; though still notably inferior to respective capital growth, in order to be in line with the results from Portico and the Valuation Office Agency. Since annual growth from local authority rents has been 4.69%, and annual capital growth has been 8.23% for the same period, it is likely that annual rental growth in the private sector is somewhere closer to 5.5%.

### Mortgages and interest rates

**Fig 3:**
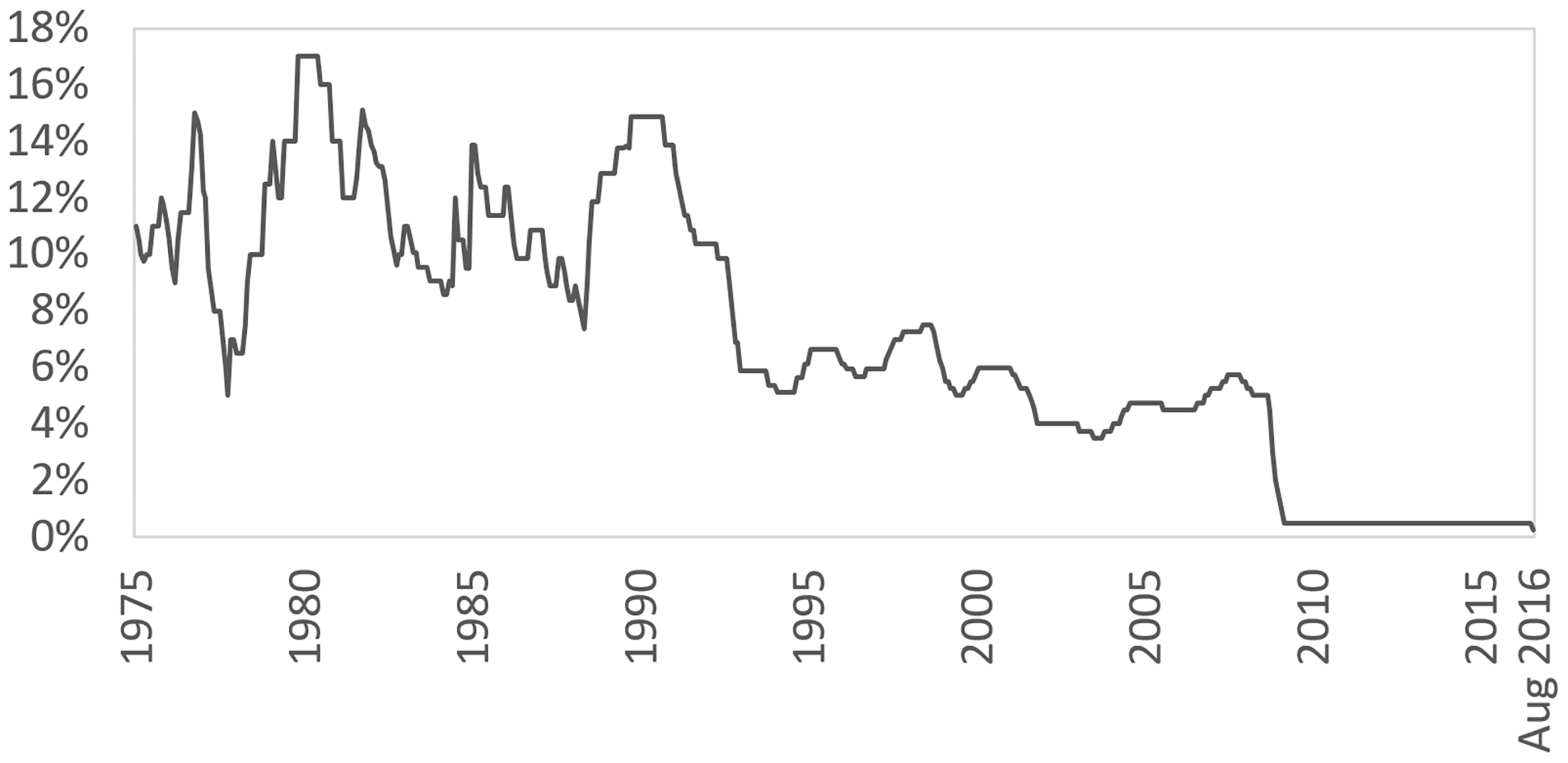
Bank of England monthly base rate records, from 1975 to date.

The interest rates offered by the various mortgage lenders in the UK are heavily influenced by the Bank of England Base Rate, which is typically used as a reference point. Data from the Bank of England [16] shows that interest rates in the UK have been at record low levels over the last few years (although this has become an international phenomenon following the financial crisis of 2007–09). Fig 3 illustrates the monthly Bank of England Base Rates records from 1975 to August 2016.

The interest rate of a mortgage is further influenced by many other factors. The most important of these currently are:

1. *Loan-To-Value (LTV) ratio*, which represents the percentage of borrowing relative to the value of the property. The higher the LTV ratio the higher the interest rate. Nowadays, LTV ratios for BTL mortgages are restricted to 75%.
2. *Arrangement fees*, which are fees you may pay the lender to set up the mortgage. These fees (currently) typically fall into three categories: 1) no fees with a relatively high interest rate, 2) fees ranging from £1,000 to £2,000 with a relatively low interest rate, and 3) fees as a percentage of the amount borrowed with a relatively low interest rate.
3. *Mortgage type*, with the most common types (both interest-only and repayment) currently being:
  - *Standard Variable Rate (SVR)*: Normally offers the highest (variable) interest rates with zero to little fees and the flexibility to repay the mortgage at any time.
  - *Trackers and Discounted Variable*: Similar to the SVR, but offer more attractive (variable) interest rates for a fixed period of time in exchange for fees and limited overpayments, with penalties if the limit is breached (the limit is typically set to 10% of outstanding mortgage balance per 12 months). The difference between a tracker and a discounted variable mortgage is that the tracker tends to follow the Bank of England’s base rate, whereas the discounted variable tends to follow the base rate set by the lender.
  - *Fixed*: Offer fixed low interest rates for a fixed period of time in exchange for fees and limited overpayments similar to (ii) above.

Note that once the agreement of a non-SVR mortgage comes to an end, which can last anywhere between 2 to 10 years (shorter durations offer lower interest rates), they revert back to the SVR. Mortgages at SVR tend to be the most expensive. A savvy property investor will typically remortgage, also known as refinancing (refer to Section 3.4), when such an agreement comes to an end.

Table 5 provides some of the most attractive mortgage deals discovered by MoneySuperMarket [17]. Note that the best mortgage is not always the one with the lowest interest rate; i.e. it depends on borrowing, fees and other factors. Further, a borrower cannot always choose the most attractive mortgage deal since each mortgage exhibits different financial and property-related requirements, and the most attractive mortgages tend to have more and stricter such requirements. Table 5 is, therefore, provided only for guidance for simulation.

**Table 5:** Some of the most attractive BTL mortgage deals (which include the option for interest only) discovered by MoneySuperMarket [17] on September 12, 2016, ranked by interest rate for different initial periods. Note that MoneySuperMarket has access to most, but not the whole, of the BTL mortgage market, and some BTL mortgage deals are only available through financial advisors. The *Fees* figure includes fees related to arrangement, admin, booking, completion, and survey. When a fee is stated as a percentage, it is calculated with reference to the amount being borrowed.

No.	Lender	Mortgage type	Initial rate	Initial period	Max LTV	Fees *(at least)*
1	Leeds	Fixed	1.65%	2 years	60%	£3,349
2	Barclays	Tracker	1.68%	2 years	60%	£1,789
3	Leek	Disc. Var.	1.99%	2 years	75%	£1,567
4	Virgin	Fixed	2.14%	2 years	70%	£2,780
5	Coventry	Fixed	2.19%	2 years	65%	£2,719
6	HSBC	Fixed	2.19%	2 years	65%	£2,671
7	Barclays	Tracker	2.2%	2 years	75%	£1,789
8	Virgin	Tracker	2.24%	2 years	70%	2.5%
9	Melton	Disc. Var.	2.19%	3 years	60%	£1,338
10	Virgin	Fixed	2.29%	3 years	60%	£2,581
11	Leek	Fixed	2.64%	3 years	75%	£1,404
12	Virgin	Fixed	2.89%	3 years	75%	£2,581
13	Leeds	Fixed	2.75%	5 years	60%	£2,499
14	Virgin	Fixed	2.78%	5 years	60%	£2,581
15	Newbury	Disc. Var.	3%	5 years	75%	£1,675
16	Virgin	Fixed	3.19%	5 years	75%	£2,581

For simulation we assume that investors tend to prefer the short-term deals which are restricted to 2-year agreements. This is because they allow investors to remortgage more frequently without penalty (i.e. every two years), and they cost less to borrowers who seek larger borrowings, which tends to be the case for BTL investors in London. Specifically, the higher the loan, the more attractive the fee to obtain a low interest rate becomes, relative to interest savings.

### Remortgage

A remortgage is the process of paying off an active mortgage through the arrangement of a new mortgage, using the same property as security. There are many reasons someone would want to remortgage. For the purpose of this paper, these include:

1. *Releasing equity*: After the initial mortgage agreement ends, the property may have increased in value. This gives the option to the investor to release equity, also known as equity withdrawal, to reinvest it in another property. This process can be seen as an aggressive form of leveraging.
  For example, consider a property bought in 2010 for £300,000 with 75% LTV; implying a deposit of £75,000. Based on an average annual capital growth of 8.5% (refer to Fig 1), the property in 2016, or after six years, is expected to be worth £489,440. Assuming an interest-only mortgage, we still owe £225,000. However, since the property is now worth much more, the revised equity is £433,742–225,000 = £264,440, which decreases the LTV rate to 45.97%. If the investor choses to increase LTV back to 75%, this would require borrowing £367,080, up from £225,000, hence releasing equity of £142,080 to reinvest in another property.
2. *Reducing LTV*: Similar to (a) above, a risk averse investor may choose to remortgage for the purpose of obtaining a lower interest rate, rather than leveraging, through the decreased LTV rate (refer to Table 5).
3. *Reducing interest rate*: Even if the property prices remain stable, an investor may still choose to remortgage to reduce the interest rate, either because the mortgage has moved to the higher SVR interest rate (refer to Section 3.3), or simply because another lender is offering a better deal.

Remortgaging still comes at a cost, however. For example, in the case of switching lenders additional mortgage fees may apply, such as mortgage broker’s and even solicitor’s fees.

### Letting agency costs

A letting agent provides lettings and full management of a BTL property, and is a popular option for investors who either have multiple properties, other full-time jobs, or who simply do not want to spend time on managing a property. In London, letting agency fees are typically more expensive than other parts of the UK. Foxtons [18] and Savills [19], who thrive in London, charge 13.2% and 15% (including VAT) relative to rental income for lettings, which involves finding tenants and arranging collection of payments, or 20.4% for full day-to-day management of the property, which extends to arranging repairs and maintenance. These fees may decrease considerably when a tenancy agreement is repeatedly renewed, as opposed to the agency having to find a new tenant. In addition to these percentage figures, there are other fees, outside of maintenance, which have potential to add up to, or above, £1,000 per annum [18, 19].

Cheaper options include smaller letting agencies which may charge a flat fee of 12% (or 10% plus VAT) of rental income, without any hidden additional fees. However, these agencies are scarce, at least throughout inner London and relative to the more popular agencies who aim to cover every major London district. Alternatively, a landlord may choose to let and manage the property without using a lettings agent. This option promises much lower costs—typically restricted to perhaps a few hundred pounds for online advertising, and some other minor expenses.

## Overview of Bayesian networks

A BN is a well-established graphical formalism that encodes the conditional probabilistic relationships amongst uncertain variables of interest. A BN model consists of nodes, which represent variables, and arcs between nodes, which represent the direction of influence [20]. Underpinning BNs is Bayesian probability inference. The term *Bayesian* comes from the Bayes’ theorem:

$$P\left(A|B\right)=\frac{P\left(B|A\right)P\left(A\right)}{P\left(B\right)}$$

which describes how to revise the prior probability of an event *A* occurring to its posterior probability conditional on new information *B*. For example, event *A* may represent a disease and factor *B* may represent a symptom. In this example, the prior probability of *A* represents the probability of having disease *A* without knowledge about symptom *B*, whereas the posterior probability of *A* represents the probability of having disease *A* given that we know whether symptom *B* is or is not present.

The structure of a BN model and resulting conditional probability tables (CTPs) can be learnt from either data or knowledge, or a combination of the two. In domains such as bioinformatics and cancer research, where the ability of experts to observe interactions between cells is limited in various ways, applying structure learning algorithms to large datasets can reveal new insights that would otherwise remain unknown. On the other hand, these structure learning algorithms become less desirable in areas where domain experts have good knowledge about the underlying causal mechanisms of the problem. Knowledge-based BN structures are popular in such cases because even the ‘best’ algorithms often make mistakes, such as ‘discovering’ that symptoms influence age. BNs are also widely recognised as the most appropriate method to model uncertainty in situations where data are limited but where human domain experts have a good understanding of the underlying causal mechanisms and/or real world facts. However, incorporating such expert knowledge into a BN model is not straightforward and hence, various methods have been proposed for this purpose [21–28].

The model presented in this paper (Section 5) has had its structure solely determined by knowledge. This is because the process of BTL is based on clearly defined rules and regulating protocols. For example, we already know that a mortgage payment is determined by the interest rate in conjunction with the amount borrowed, and that tax payments are determined based on specific thresholds associated with different amounts of income. There is no need to collect relevant data about these factors and apply structure learning methods in an effort to ‘discover’ these relationships.

**Fig 4:**
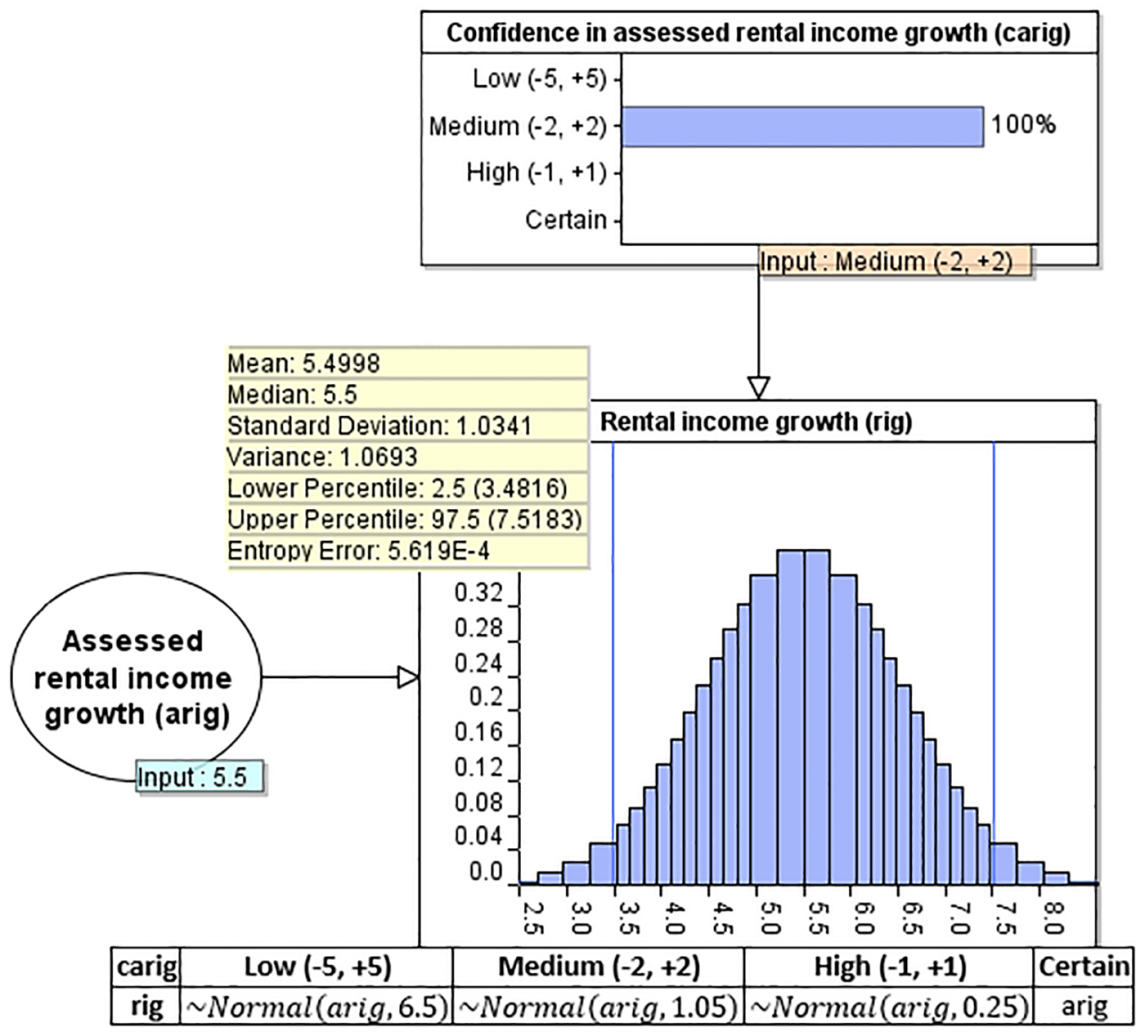
A simple 3-node BN, with the CPT of node *rental income growth* and summary statistics superimposed in the graph.

Fig 4 presents a simple 3-node BN, which consists of both discrete and continuous variables. BN models which incorporate both discrete and continuous variables are often referred to as *hybrid* BNs. This example demonstrates how we incorporate and quantify uncertainty in the BTL process. It shows how the subjective indication of 5.5% about the growth rate of the rental income is assessed in conjunction with deterministic degrees of confidence (in this case *Medium* which generates ±2% at 95% CI). The CPT and summary statistics of the *Rental income growth* distribution are superimposed in the graph. We have made use of the AgenaRisk BN software [29] which allows simulation of continuous distributions using Dynamic Discretisation [30]. The same parameter learning process applies to a number of other hypothetical inputs. We cover these in detail in Section 5.

When it comes to time-series analysis with BNs, there are two main approaches [31]. The first approach is widely known as a *dynamic* BN (DBN), which represents a single model that captures all of the properties of the system dynamics and which is capable of producing distributions that relate to different trajectories. DNBs are also generalisations of hidden Markov models [32]. The second approach, which is not as compact as the first one, enables us to relate variables to each other over adjacent time steps without being restricted to a single model that captures all of the properties of the system. For example, a temporal BN (TBN) allows for non-identical networks to exist at each time step. Note that *Gated* BNs (GBNs) are similar to TBNs in the sense that they represent processes which include several distinct phases, but GBNs tend to distinguish between sub-networks that are activated or disabled under specific scenarios. Application areas concerned with aspects of time-series analysis with BNs include the application of DBNs to agricultural developments [33], forensic psychiatry [34], medicine [35, 36], and bioinformatics [37]; the application of GBNs to algorithmic trading and stock market movements [38], and the application of TBNs to predicting long-term football team performance [39]. It should be noted that, while we focus on the probabilistic prediction approach for TBNs in this paper, recent theoretical work uses similar ideas to explore the structure and behaviour of complex systems. For example, [40] introduced *forest likelihood* to assess the complexity of such sparse networks, where the likelihood of the appearance of each forest can be analytically calculated within the framework.

In general, BNs have become increasingly popular in various real-world areas which requires improved decision making processes, including medicine [41–43], project management [33, 44], forensics and legal reasoning [45–48], finance and marketing [49], sports [50], and software engineering [51, 52]. In the property market domain, studies tend to focus on house sale trends using Bayesian estimation and/or averaging. Some of these studies include analyses of: the housing market dynamics by the European Central Bank [53]; the Colombian house price data [54]; what drives Ireland’s housing market [55]; the residential property valuation in Hong Kong [56]; downturns [57] and repeat sales indices [58] in US housing market; house sales price in Toronto [59]; the factors which capitalise into house prices in Zurich [60]. In contrast to these past relevant publications, we focus on the property market from the investor’s perspective. We examine the impact the new tax measures have on the profitability of the typical BTL property in London, and assess the relevant risks and potential profits of a BTL investment under the pending tax reforms. We have developed a bespoke TBN for this purpose.

## The model

**Fig 5:**
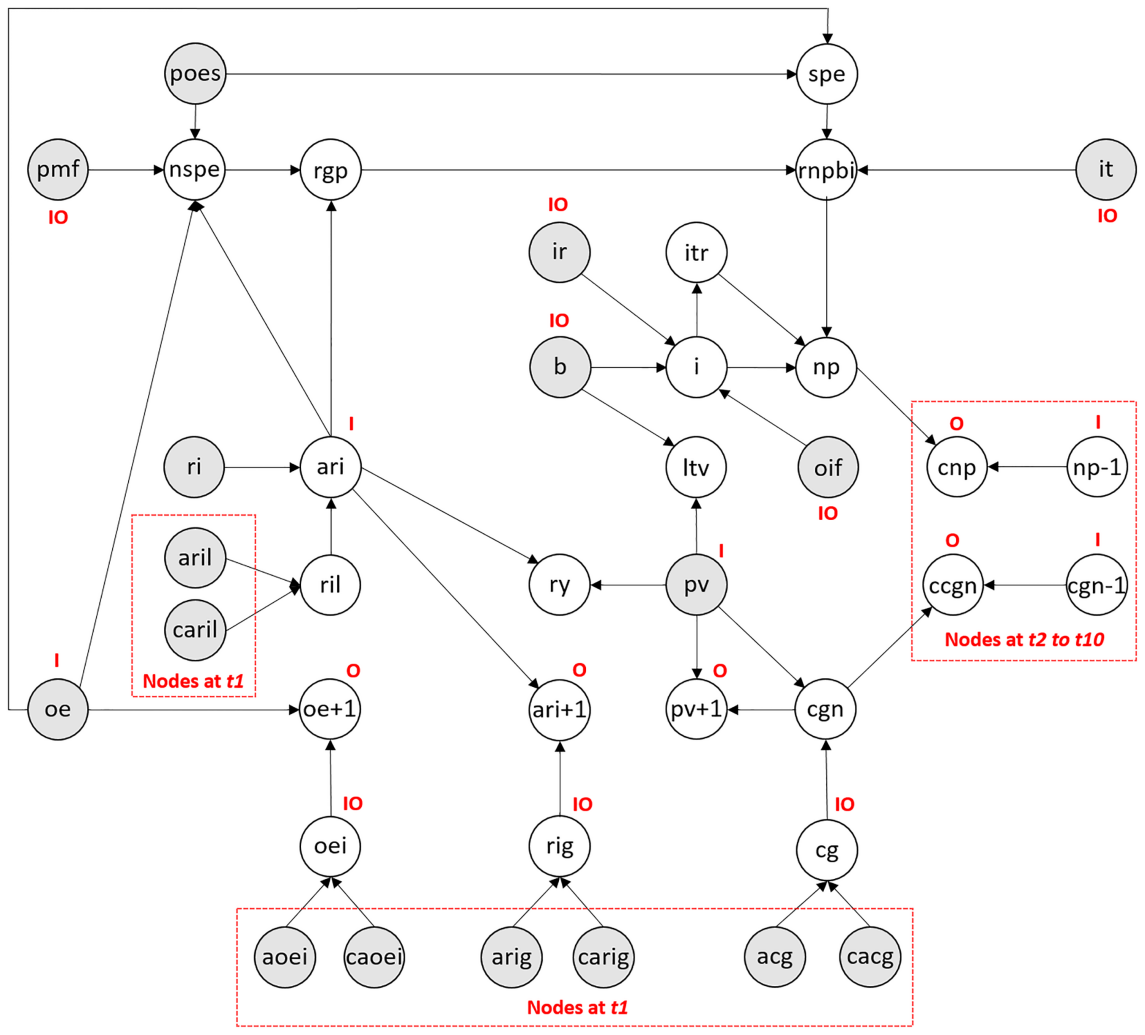
Topology of the TBN model, where a shaded node is an observable variable, a white node is a latent variable, *I* is input node, *O* is output node, and *IO* is both an input and an output node.

The topology of the overall TBN is presented in Fig 5. A full description for each of the nodes and respective CPTs is provided in Table 6. The shaded nodes in Fig 5 represent *background* nodes. A background node is simply a variable that has no ancestors and, for the purposes of this simulation study, represents required input such as property value, interest rate, borrowing, and so on. Note that, while nodes *np-1* and *cgn-1* in Fig 5 have no ancestors, they only exist in time periods *t2* to *t10* and hence are not background nodes since they are dependent on *t1*. Fig 5 also distinguishes the following types of nodes:

1. The nodes which are restricted to specific time periods. The model assumes that a single time period represents a single year and hence, *t5* represents year five and *t+1* implies one year after period *t*. Restricting the representation of a single time period *t* to a whole year, rather than days or months, not only allowed us to maintain the complexity of the model at minimum levels, but also to build a reasonably accurate knowledge-based system of the BTL process which is driven by annual regulations and figures.
2. The input (I) and output (O) nodes. A BN model exists for each time period *t*, and the input and output nodes are the nodes which connect those BN models within the temporal space. Specifically, an input node at time *t* takes as input (i.e. prior distribution) the respective output (i.e. posterior distribution) associated with this node at time *t-1*. Note that, since *t1* represents the starting time interval, input nodes at *t1* serve as the background nodes, as discussed above, which require hypothetical observations entered for simulation purposes (refer to Section 5.1). For example, the input node *Property value* (*pv*) at time *t* takes as input the output of node *pv at t-1*.

**Table 6:** Description of each of the TBN model nodes as defined in the topology of Fig 5.

Variable type	BN variable	Description	CPT
*Observable*	*acg*	Assessed capital growth	[−∞, ∞]
*Observable*	*aoei*	Assessed other expenses inflation	[−∞, ∞]
*Observable*	*arig*	Assessed rental income growth	[−∞, ∞]
*Observable*	*aril*	Assessed rental income loss	[0, ∞]
*Observable*	*b*	Borrowing	[0, ∞]
*Observable*	*cacg*	Confidence in assessed capital growth	L*ow* = *Medium* = *High* = *Certain* = 0.25
*Observable*	*caril*	Confidence in assessed rental income loss	L*ow* = *Medium* = *High* = *Certain* = 0.25
*Observable*	*caoei*	Confidence in assessed other expenses inflation	L*ow* = *Medium* = *High* = *Certain* = 0.25
*Observable*	*carig*	Confidence in assessed rental income growth	L*ow* = *Medium* = *High* = *Certain* = 0.25
*Observable*	*ir*	Interest rate	[0, ∞]
*Observable*	*it*	Income tax	[0, ∞]
*Observable*	*oe*	Other expenses (e.g. service charges, ground rent, broker fees)	[0, ∞]
*Observable*	*oif*	Other interest-related fees	[0, ∞]
*Observable*	*pmf*	Property management fees	[0, ∞]
*Observable*	*poes*	% of property expenses as servicing	[0, ∞]
*Observable*	*pv*	Property value	[0, ∞]
*Observable*	*ri*	Rental income	[0, ∞]
*Latent*	*ari*	Actual rental income	$ri\times \frac{\left(1-ril\right)}{100}$
*Latent*	*ari+1*	Actual rental income at *t+1*	$ari+ari\times \frac{rig}{100}$
*Latent*	*ccgn*	Cumulative capital gains	*cgn* + *cgn* − 1
*Latent*	*cg*	Capital growth	$f\left(cg\right)=\{\begin{array}{ll} \sim Normal\left(acg,\;6.5\right), & \;cacg="Low"\\ \sim Normal\left(acg,\;1.05\right), & \;cacg="Medium"\\ \sim Normal\left(acg,\;0.25\right), & \;cacg="High"\\ acg, & \;cacg="Certain" \end{array}$
*Latent*	*cgn*	Capital gains	$\frac{cg}{100}\times pv$
*Latent*	*cgn-1*	Capital gains at *t-1*	*cgn* at *t-1*
*Latent*	*cnp*	Cumulative net profit	*np* + *np* − 1
*Latent*	*i*	Interest	$\frac{b\times ir}{100}+oif$
*Latent*	*itr*	Interest tax relief	*min*(*pv*_*t*1_, *i*) × 0.2
*Latent*	*ltv*	Loan to value (LTV)	$\frac{b}{pv}$
*Latent*	*nspe*	Non-servicing property expenses	$\frac{ari\times pmf}{100}+oe\times \left(1-ppes\right)$
*Latent*	*oei*	Other expenses inflation	$f\left(oeg\right)=\{\begin{array}{ll} \sim Normal\left(aoeg,\;6.5\right), & \;caoeg="Low"\\ \sim Normal\left(aoeg,\;1.05\right), & \;caoeg="Medium"\\ \sim Normal\left(aoeg,\;0.25\right), & \;caoeg="High"\\ aoeg, & \;caoeg="Certain" \end{array}$
*Latent*	*oe+1*	Other expenses at *t+1*	$oe+oe\times \frac{oeg}{100}$
*Latent*	*pv+1*	Property value at *t+1*	*pv* + *cgn*
*Latent*	*rgp*	Rental gross profit	*ari* − *pe*
*Latent*			
*Latent*	*rig*	Rental income growth	$f\left(rig\right)=\{\begin{array}{ll} \sim Normal\left(aril,\;6.5\right), & \;caril="Low"\\ \sim Normal\left(aril,\;1.05\right), & \;caril="Medium"\\ \sim Normal\left(aril,\;0.25\right), & \;caril="High"\\ aril, & \;caril="Certain" \end{array}$
*Latent*	*ril*	Rental income loss	$f\left(ril\right)=\{\begin{array}{ll} \sim Normal\left(aril,\;6.5\right), & \;caril="Low"\\ \sim Normal\left(aril,\;1.05\right), & \;caril="Medium"\\ \sim Normal\left(aril,\;0.25\right), & \;caril="High"\\ aril, & \;caril="Certain" \end{array}$
*Latent*	*rnpbi*	Rental net profit before interest	$rgp\times \left(1-\frac{it}{100}\right)-spe$
*Latent*	*ry*	Rental yield	$\frac{ari}{pv}\times 100$
*Latent*	*spe*	Servicing property expenses	*oe* × *pps*
*Latent*	*np*	Net profit	*itr* + *rnpbi* − *i*
*Latent*	*np-1*	Net profit at *t-1*	*np* at *t-1*

The TBN model incorporates a number of subjective nodes which allow the model to capture the investor’s uncertainty relating to the inflation in property expenses, rental income growth, rental income loss from void periods, and capital growth. For each of these cases, we have introduced an *Assessed* node, which takes as input the investor’s belief about the given rate, and a *Confidence* node, which takes as input the investor’s degree of confidence with respect to the input rate, as previously illustrated in the example BN model of Fig 4. Further, Table 6 shows that the available states of a *Confidence* node are *Low*, *Medium*, *High*, or *Certain*, and each state generates distributions with the respective deterministic variabilities of ±5%, ±2% ±1% and ±0%, at 95% confidence interval (CI). These observable nodes exists only at *t1*, as discussed above and also shown in Fig 5.

It is important to note that the BN model described in this paper is developed for simulation purposes based on different hypothetical BTL investment scenarios. These scenarios are based on different hypothetical observations related to various factors of interest such as interest rate, capital and rental growth, along with other relevant factors (refer to Section 6). The purpose of the simulation is to demonstrate how hypothetical changes in these factors may influence a BTL investment, and *not* to predict how these factors may change in the future. For example, while it is reasonable to assume that capital and rental growth are positively correlated, there is no need to incorporate such an assumption into this model since we do not seek to predict these outcomes, but we rather assign hypothetical observations to them.

Finally, throughout an investment period an investor may want to intervene on a BTL investment for various reasons, such as to revise borrowing, renew interest rate deals, etc. The causal interpretation underpinning BNs makes these models particularly suitable in informing how outcomes would change as a result of an intervention. Most previous work on BN interventions focus on interventions performed on a particular state of a node that is made independent of all its ancestors. This process is known as *graph surgery* [20] and represents the standard process for interventional analysis in BNs. In our case, we are interested in intervening on a particular state of a node at a particular time period; hence, making an event at *t* independent of *t-1*. As a result, we perform *graph surgery* between time steps, or between BN models within the temporal space, rather than between nodes in a single BN model.

## Simulation and results

In order to examine the influence of various relevant factors on a BTL investment, we determine a BTL profile. This profile, which we refer to as the Typical BTL London Profile (TBLP), is intended to represent a common profile for BTL property investments in London. The profile serves as the basis for assessment of each of the factors of interest and their impact on the profitability of the investment profile. The description of the TBLP is provided in Section 6.1. Subsequent subsections demonstrate the results from simulation for each of the assessed factors.

### The Typical BTL London Profile (TBLP)

The TBLP is determined by the status of the property market in 2016. The observable input values considered for this profile are provided in Table 7. These inputs are primarily based on what has been discussed in Section 3, as well as on some additional assumptions. The TBLP is determined from the following assumptions or facts:

1. According to Land Registry [8], the average property sale in London in 2016 (up to August) was £635,582, and the median £430,000. We consider the purchase of a property at £500,000.
2. The typical BTL investor seeks to purchase a property with maximum borrowing, or minimum deposit. As a result, we consider that the LTV is set to the current market available maximum of 75% (i.e. £375,000 borrowing). Note that, as of May 2017, new regulations on rental stress tests have made deposits at 25% largely insufficient, although occasionally lenders may consider other affordability figures in determining the maximum LTV (see Section 6.2 for more details).
3. We consider an interest rate of 2.2% at 75% LTV, and an interest rate of 1.7% at 60% LTV or lower, plus an average of £1,000 fees per annum (e.g. arrangement and valuation fees averaged per annum). These inputs are based on what has been discussed in Sections 3.3 and 3.4, and using Table 5 as guidance. Note that while most lenders allow arrangement fees to be added to the loan, rather than pay them upfront, they still represent a fee and which we consider as an upfront cost when calculating net profit from rental income.
4. We consider a rental yield of 4% (i.e. £20,000) based on the statistics retrieved by [12], and an annual rental income growth of 5.5% (with *Medium* confidence), based on what has been discussed in Section 3.2. We also assume that the loss in rental income from void periods in London averages to two weeks per annum (i.e. 3.85% loss from rental income), with *Medium* confidence.
5. We assume that the BTL investment is fully managed by a lettings agency. Further to what has been discussed in Section 3.5, while the bigger letting agencies in Inner London charge 20.4% (including VAT) of rental income, numerous other smaller agencies, particularly those based in Outer London, charge 12%. We have not found accurate statistics on this matter, hence we consider the management fees to be 16% (including VAT), which represents the approximate middle point of the range available in the market.
6. The *Other property expenses* figure is highly volatile since it may include service charges, ground rent, maintenance costs, solicitor’s fees from remortgages, plus any other additional fees a lettings agent may charge and which fall outside of the fixed percentage charge. We assume this figure to be £2,500 per annum, 20% of which (or £500) falls under the ‘servicing’ category and which cannot be used for tax allowance purposes (refer to Section 2.3). We also assume that these expenses grow by 5% per annum (i.e. faster than typical inflation which we cover later in Section 7.2), with *Low* confidence.
7. We consider a 10-year average annual capital growth of 8.5%, with *Low* confidence, based on what has been discussed in Section 3.1.
8. We assume the landlord falls within the *High rate* income tax band of 40% (see Table 1).

**Table 7:** The Typical BTL London Profile (TBLP) considered for simulation.

Factor (BN variable)	Value	Confidence level
Property (purchase) value (*pv*)	£500,000	n/a
Borrowing (*b*)	£375,000	n/a
Interest-only rate at 75/60% LTV (*ir*)	2.2/1.7%	n/a
Other (annual) interest-related fees (*oif*)	£1,000	n/a
(Annual) Rental income (*ri*)	£20,000	n/a
(Annual) Rental income loss (*aril* & *caril*)	3.85%	Med. (±2%)
Property management fees (*pmf*)	16%	n/a
Other (annual) expenses (*oe*)	£2,500	n/a
% of other expenses as servicing (*poes*)	20%	n/a
Other expenses inflation (*aoei* & *caoei*)	5%	Low (±5%)
Rental income growth (*arig* & *carig*)	5.5%	Med. (±2%)
Capital growth (*acg* & *cacg*)	8.5%	Low (±5%)
Income tax (*it*)	40%	n/a

Table 8 illustrates the results from simulation based on the inputs presented in Table 7, and under the assumption that the interest-only mortgage is based on 2-year fixed-rate agreement, which is renewed every two years without releasing equity. At this stage, the results reported are restricted to the expected values of the distributions. Further, since almost all mortgage lenders in the UK allow for annual overpayments up to 10% of residual mortgage balance, if we were to reinvest net profits from rental income generated at the end of each time period as mortgage overpayments, this would have increased the total net returns by £1.5k due to the reduced interest payments. Because of the minor impact and for simplicity, we shall assume that the typical investor saves net profits for cash flow.

**Table 8:** Results (expected values where applicable) from simulation based on the TBLP of Table 7. Monetary values represent £GBPs in thousands (k). The *Real rental income/yield* figures represent the rental income/yield after accounting for potential rental income loss due to void periods.

*t*	Real rental income	Real rental yield	Property expenses (overall)	Interest & fees	Net profit	Gross capital gains	Property value at *t+1*	LTV at *t+1*
*1*	19.2k	3.8%	5.6k	9.25k	0.6k	42.5k	542.5k	69.1%
*2*	20.3k	3.7%	5.9k	9.25k	1k	46.1k	588.6k	63.7%
*3*	21.4k	3.6%	6.2k	9.25k	1.5k	50k	638.7k	58.7%
*4*	22.6k	3.5%	6.5k	9.25k	2k	54.3k	692.3k	54.2%
*5*	23.8k	3.4%	6.9k	7.38k	4k	58.9k	751.9k	49.9%
*6*	25.1k	3.4%	7.2k	7.38k	4.6k	63.9k	815.8k	46.0%
*7*	26.5k	3.3%	7.6k	7.38k	5.2k	69.3k	885.1k	42.4%
*8*	28k	3.2%	8k	7.38k	5.8k	75.2k	960.4k	39.0%
*9*	29.5k	3.1%	8.4k	7.38k	6.5k	81.6k	1042k	36.0%
*10*	31.1k	3%	8.9k	7.38k	7.5k	88.6k	1130.6k	33.2%

### Impact of rental yield

Table 9 illustrates the impact rental yield has on net profits. The results show that a change of a single percentage point in rental yield has an impact of ±£31.2k on cumulative net profits, over the 10 years. At 2.8% rental yield, and with confidence set to ±2% at 95% CI, the TBLP has a risk of loss 11.4% (excluding capital gains), and this risk increases to 100% when rental yield decreases further to 2.6%.

**Table 9:** The impact of rental yield on 10-year cumulative net profits. Monetary values represent £GBPs in thousands (k). The highlighted row represents the point at which the investment has risk of becoming lossmaking.

Rental yield	Rental yield relative to TBLP	Net profit (95% CI)	Risk of loss	Net profit relative to TBLP (*EV*)
2%	-2%	[-25.2k, -22.8k]	100%	-62.4k
2.5%	-1.5%	[-9.9k, -6.9k]	100%	-47.1k
2.6%	-1.4%	[-6.8k, -3.8k]	100%	-44k
2.7%	-1.3%	[-3.7k, -0.6k]	99.6%	-40.9k
**2.8%**	**-1.2%**	**[-0.6k, 2.6]**	**11.4%**	**-37.7k**
2.9%	-1.1%	[2.5k, 5.7k]	0%	-34.6k
3%	-1%	[5.5k, 8.9k]	0%	-31.2k
4%	-	[36.3k, 40.5k]	0%	-
5%	1%	[67.1k, 72.2k]	0%	+31.2k
6%	2%	[97.8k, 103.9k]	0%	+62.4k
7%	3%	[128.5k, 135.5k]	0%	+93.6k
8%	4%	[159.3k, 167.2k]	0%	+124.8k

However, the impact of rental yield extends to much more significant issues. This is because mortgage lenders use rental yield as the basis for assessing the amount they can lend. The proportion of rental income to mortgage interest payments is also known as debt service coverage ratio. Currently, lenders require that a BTL property generates gross rental income that is at least 125–165% of the mortgage payments (note that as of May 2017, rental stress-tests require a minimum of 140%). However, instead of taking the actual mortgage payments into account, lenders stress-test this risk based on high-risk hypothetical interest rates, currently ranging between 5% and 6%. In the case of borrowing £375k, a lender with 125% rental coverage requirements at 5% interest rate would want the property to generate a monthly rental income equivalent to $\frac{£375.000\;\times 0.05}{12}\times 1.25=£1953.13$, which represents a rental yield of 4.69%.

Tougher requirements of 145% rental yield at 5.5% interest rate increase rental yield requirements substantially, pushing the acceptable rental yields well above the current average observed in London. However, some lenders may relax some of the stress-test requirements for experienced BTL investors and occasionally, they may consider other forms of income to help borrowers meet affordability demands. Otherwise, the only option left to a borrower is to reduce LTV down to a particular threshold so that rental income passes the stress-test.

### Impact of letting agency fees

In Section 3 we mentioned that some letting agencies will occasionally charge additional fees that fall out of the fixed percentage fee. The TBLP assumes these fees to be £250 per annum (this figure is incorporated into *Other expenses* (*oe*)), with the fixed percentage letting agency fee set to 16% inc VAT. In simulating the impact of letting agency fees, we manipulate both of these values. The results in Table 10 show that an investor who does not hire a letting agent is expected to increase net profits by £34.5k, over the 10-year period, relative to an investor who hires one of the more expensive letting agents for full management of the property.

**Table 10:** The impact of letting agency fees on 10-year cumulative net profits. Monetary values represent £GBPs in thousands (k).

Agent fees	Agent fees relative to TBLP	Changes in other fees	Net profit (95% CI)	Risk of loss	Net profit relative to TBLP (*EV*)
0%	-16%	-£250	[61.9k, 66.8k]	0%	+25.9k
5%	-11%	-£250	[54.6k, 59.3k]	0%	+18.5k
5%	-11%	£0	[52.4k, 57.1k]	0%	+16.3k
10%	-6%	-£250	[47.2k, 50.7k]	0%	+10.6k
10%	-6%	£0	[45k, 49.6k]	0%	+8.9k
13%	-3%	-£250	[42.9k, 47.2k]	0%	+6.7k
13%	-3%	£0	[40.6k, 45k]	0%	+4.5k
16%	0%	£0	[36.3k, 40.5k]	0%	-
18%	2%	£0	[33.3k, 37.5k]	0%	-3k
18%	2%	+£250	[31.1k, 35.4k]	0%	-5.1k
20.4%	4.4%	£0	[29.8k, 33.9k]	0%	-6.5k
20.4%	4.4%	+£250	[27.7k, 31.8k]	0%	-8.6k

### Impact of interest rates

In Section 6.1 we explained how the TBLP considers the interest rates of 2.2% and 1.7%, depending on LTV; i.e. interest rates decrease to 1.7% if LTV decreases to 60%. Table 8 shows that the requirements for the lower interest rate are met at *t3*. However, since the mortgage is based on a 2-year fixed rate deal, the lower interest rate can be obtained at the end of *t4* when the 2^nd^ remortgaging stage occurs (assuming equity is not released). As a result, simulation assumes an interest rate of 2.2% for time periods *t1* to *t4*, and an interest rate of 1.7% for time periods *t5* to *t10*. The results in Table 11 show that a change of a single percentage point in interest rates has an impact of ±£30k on cumulative net profits. If interest rates were to rise by an absolute 1.2%, the TBLP investment would have a risk of 1.3% for loss, and this risk becomes 100% if interest rates were to rise by 1.5%.

**Table 11:** The impact of interest rates on 10-year cumulative net profits. Monetary values represent £GBPs in thousands (k). The highlighted row represents the point at which the investment has risk of becoming lossmaking.

Initial interest rate (t1 –t4 at 75% LTV)	Successive interest rate (t5 –t10 at ≤60% LTV)	Interest rate relative to TBLP	Net profit (95% CI)	Risk of loss	Net profit relative to TBLP (*EV*)
1.7%	1.2%	-0.5%	[51.3k, 55.5k]	0%	+15k
1.95%	1.45%	-0.25%	[43.9k, 48k]	0%	+7.5k
2.2%	1.7%	-	[36.4k, 40.5k]	0%	-
2.45%	1.95%	+0.25%	[28.8k, 33k]	0%	-7.5k
2.7%	2.2%	+0.5%	[21.3k, 25.5k]	0%	-15k
2.95%	2.45%	+0.75%	[13.9k, 18k]	0%	-22.5k
3.2%	2.7%	+1%	[6.3k, 10.6k]	0%	-30k
3.3%	2.8%	+1.1%	[3.3k, 7.6k]	0%	-33k
**3.4%**	**2.9%**	**+1.2%**	**[0.3, 4.5]**	**1.3%**	**-36k**
3.5%	3%	+1.3%	[-2.7k, 1.5k]	70.9%	-39k
3.6%	3.1%	+1.4%	[-5.7k, -1.4k]	99.9%	-42k
3.7%	3.2%	+1.5%	[-8.7k, -4.5k]	100%	-45k
4.2%	3.7%	+2%	[-23.7k, -19.5k]	100%	-60k
5.2%	4.7%	+3%	[-53.6k, -49.5k]	100%	-90k
6.2%	5.7%	+4%	[-83.7k, -79.5k]	100%	-120k
7.2%	6.7%	+5%	[-113.7k, -109.5k]	100%	-150k

### Impact of capital growth

Annual capital growth is one of the more volatile factors being simulated. While we consider the average annual capital growth, over a 10-year period, to be 8.5% (refer to Section 3.1), data from [8, 9] show that for any 10-year period since 1973, the minimum and maximum averaged capital growth rates in London range between 2% and 18% respectively. As shown in Table 12, at 2% average annual growth, and with confidence set to ±5% at 95% CI, the TBLP has risk 0.7% for loss, and this risk increases to 100% when the capital growth decreases by 4% per annum.

**Table 12:** The impact of capital growth on 10-year cumulative capital gains. Monetary values represent £GBPs in thousands (k). The highlighted row represents the point at which the investment has risk of becoming lossmaking.

Capital growth	Capital growth relative to TBLP	Gross capital gains relative to TBLP (*EV*)	Gross capital gains (95% CI)	Risk of loss
-5%	-13.5%	-831.3k	[-265.8k, -136.1k]	100%
-4%	-12.5%	-798.1k	[-235.1k, -100.2k]]	100%
-3%	-11.5%	-761.9k	[-201.3k, -61.5k]	99.9%
-2%	-10.5%	-722k	[-164.7k, -18.2k]	99.3%
-1%	-9.5%	—678.3k	[-124k, 28.4k]	89.1%
0%	-8.5%	-630.6k	[-79.9k, 79.9k]	50%
1%	-7.5%	-578.2k	[-31.1k, 135.7k]	10.9%
**2%**	**-6.5%**	**-521.2k**	**[21.9k, 197.1k]**	**0.7%**
3%	-5.5%	-459.6k	[79.9k, 264.2k]	0%
4%	-4.5%	-390.5k	[143.4k, 337.3k]	0%
5%	-3.5%	-316k	[213k, 416.8k]	0%
6%	-2.5%	-235.2k	[288k, 503.5k]	0%
7.5%	-1%	-100.1k	[414.4k, 647.6k]	0%
8.5%	-	-	[507.6k, 755k]	0%
9.5%	1%	108.6k	[609.9k, 870.2k]	0%
11%	2.5%	289.1k	[777.8k, 1063.8k]	0%
13%	4.5%	565.4k	[1036.7k, 1360.7k]	0%
15%	6.5%	892.3k	[1341.2k, 1708.7k]	0%
18%	9.5%	1486.5k	[1897.2k, 2342.8k]]	0%
20%	11.5%	1965.4k	[2345.7k, 2853.4k]	0%
25%	16.5%	3526.3k	[3809k, 4517k]	0%

Table 12 also indicates that a single percentage point difference in capital growth has highly volatile impact. For example, if we were to experience a decrease or increase of one percentage point on the annual average capital growth of 2%, the expected impact would have been -£57k and +£61.6k respectively. However, when the annual capital growth averages at 18% per annum the respective impact is -£213.6k and +£229.1k. The rule of thumb is that the value of the property doubles every 10 years if the annual capital growth averages roughly at 7.5%.

### Impact of leverage

When property prices increase, an investor may choose to release equity to reinvest it in another property. This is a common investment direction for BTL investors who favour leveraging. However, any additional borrowing above the capital value of the property when it was brought into the letting business is not tax deductible (see *itr* in Table 6). Further, the process of leveraging entails greater uncertainties than those in preceding scenarios. To ensure the simulation is realistic, we restrict the value of equity that can be released at each remortgaging stage to roughly a level that ensures additional interest paid from increased borrowing does not result in negative net profit at 99% CI. In the case where the 99% mass of the distribution falls into negative net profit, for any time *t*, the LTV rate is decreased in steps of 5 percentage points until this condition is met. This restriction inflates the proportional minimum deposit required when buying additional properties through simulation, and this makes simulation more realistic and in line with the expected stricter affordability stress-tests.

Table 13 indicates the equity that can be released at each remortgaging stage, which we assume occurs every two years, as indicated in Section 6.1. Note that in order to satisfy the requirement on net profits, the LTV is restricted to 70% at the third and fourth remortgaging stages (i.e. *t6* and *t8*), and to 65% at the fifth remortgaging stage (which may occur at the end of the 10-year period), down from current obtainable maximum of 75%.

**Table 13:** Equity that can be released at each remortgaging stage, with the restriction that additional borrowing does not lead to negative net profit at 95% CI for any time *t*. Monetary values represent £GBPs in thousands (k).

*t*	Property value at *t*	Deposit at *t*	Borrowing at *t*	LTV at *t*	Property value at *t+1*	Equity released at *t+1*	LTV at *t+1*	Interest and fees	Net profit (*EV*)
*t1*	500k	125k	375k	75%	542.5k	-	69.1%	9.3k	0.6k
*t2*	542.5k	167.5k	375k	69.1%	588.6k	66.5k	75%	9.3k	1k
*t3*	588.6k	147.2k	441.5k	75%	638.7k	-	69.1%	10.7k	0.3k
*t4*	638.7k	197.3k	441.5k	69.1%	692.3k	77.8k	75%	10.7k	0.8k
*t5*	692.3k	173.1k	519.2k	75%	751.9k	-	69.1%	12.4k	-0.1k
*t6*	751.9k	232.7k	519.2k	69.1%	815.8k	51.8k	70%	12.4k	0.5k
*t7*	815.8k	244.7k	571.1k	70%	885.1k	-	64.5%	13.6k	-0.1k
*t8*	885.1k	314k	571.1k	64.5%	960.4k	101.2k	70%	13.6k	0.5k
*t9*	960.4k	288.1k	672.3k	70%	1042.2k	-	64.5%	15.8k	-1.0k
*t10*	1042.2k	369.9k	672.3k	64.5%	1130.9k	62.8k	65%	15.8k	-0.3k

**Fig 6:**
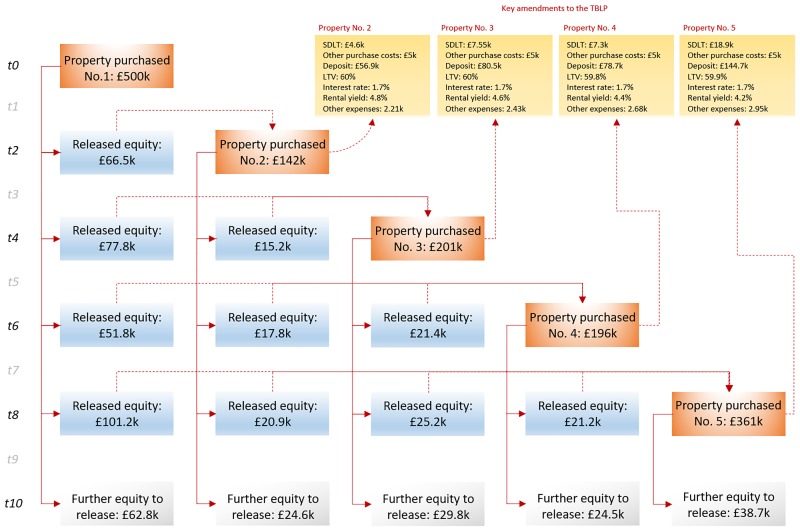
The process of leveraging by increasing borrowing through gross capital gains, and using released equity to fund the purchase of additional properties.

Fig 6 illustrates how the equity released at each remortgaging stage allows investors to fund further BTL properties throughout the 10-year period, which in turn may provide the option to release additional equity that can be used for the same purpose. For each additional property purchased, a number of key amendments are reported with respect to the TBLP. Note that each additional property is purchased at 60% LTV with the lower interest rate of 1.7%, as a result of the restriction introduced on net profits.

The outcomes of Fig 6 are based on the following facts or assumptions:

1. When calculating the available deposit to fund a new BTL investment, we factor in the costs from the new SDLT (refer to Section 2.2), plus a generous £5k to cover other property purchase costs (e.g. land registry and solicitor fees).
2. Each additional property purchased is assumed to have rental yield that is 1% higher relative to the TBLP. This is because the additional properties purchased are much cheaper and hence, more likely to be studios and 1-bedroom flats which tend to generate higher rental yields. The simulation also accounts for the annual projected decrease of 0.1% in rental yields, to be in agreement with the results in Table 8. For example, Fig 6 shows that at *t2 Property No*.*2* starts with rental yield 4.8% (i.e. 4% + 1% − 0.2%), whereas at *t4 Property No*.*3* starts with rental yield 4.6% (i.e. 4% + 1% − 0.4%).
3. The *Other expenses* figure (refer to Section 6.1) is assumed to be £500 lower relative to the TBLP, since properties of lower value tend to entail lower such expenses. However, simulation also accounts for the 5% annual inflation rate (refer to Table 7), in the same way it does for the decreasing rental yield. For example, Fig 6 shows that at *t2 Property No*.*2* has initial *Other expenses* set to £2.21k (i.e. (*£*2.5*k* − 0.5*k*) × 1.05^2^), whereas at *t4 Property No*.*3* has £2.43k (i.e. (*£*2.5*k* − 0.5*k*) × 1.05^4^).

**Fig 7:**
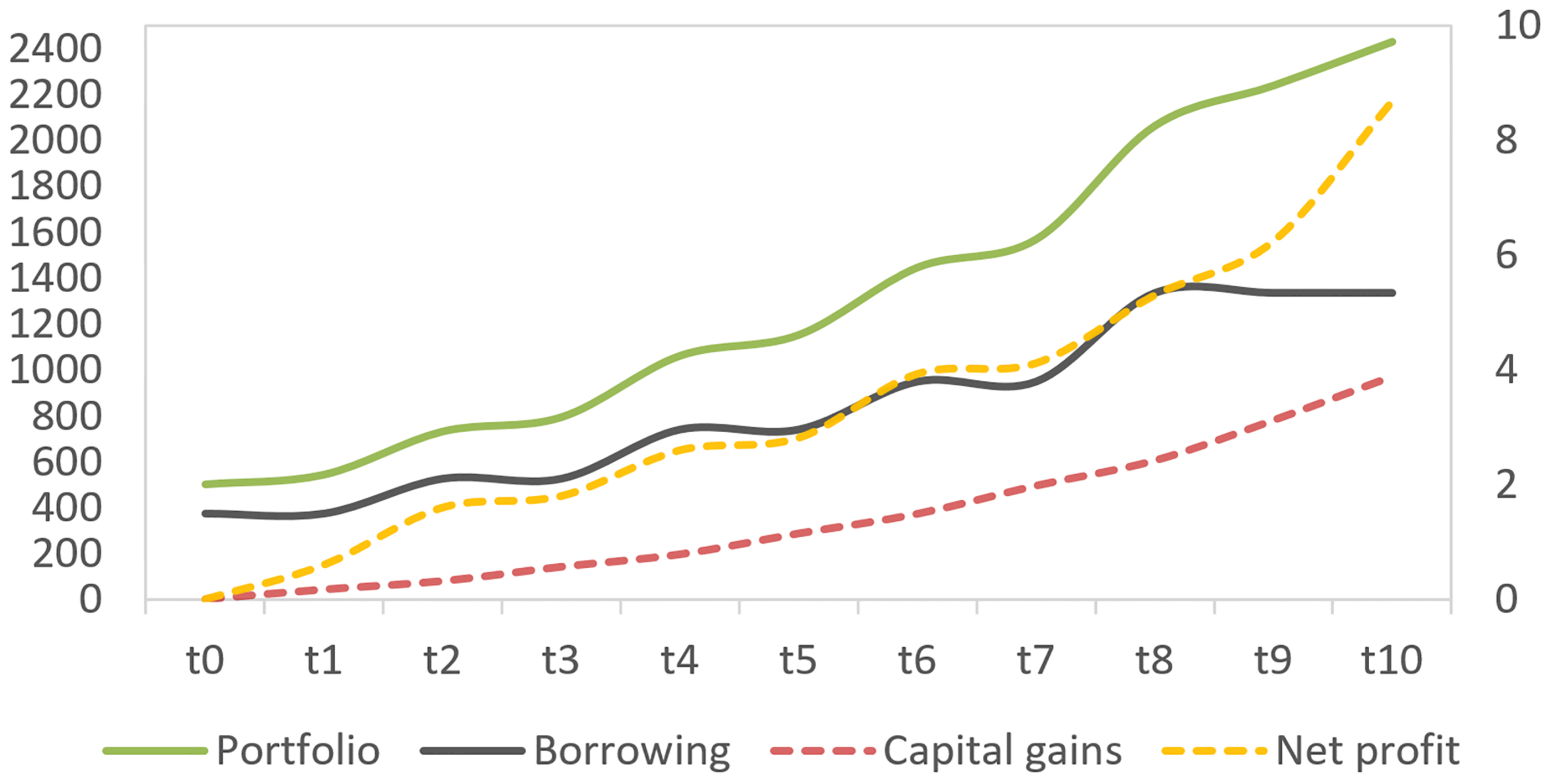
Cumulative portfolio, gross capital gains and borrowing (left axis), and net profit (right axis), as a result of leveraging. Both left and right axis represent £GBPs in thousands (k).

Fig 7 demonstrates the cumulative property portfolio, borrowing, gross capital gains, and net profit, as a result of leveraging, by taking all of the five prospective properties into consideration as illustrated in Fig 6. Note that leverage stops after *t8* without accounting for the equity that could have been released at the end of *t10* to buy a sixth property.

## Discussion of the results

In Section 6 we demonstrated the impact various key factors have on the profitability of the TBLP. These results are discussed in Section 7.1. Profitability comparisons between the old and new tax reforms are provided and discussed in Section 7.2.

### Factors of interest and their impact on the TBLP

There are factors outside of the investor’s control which can impact net profits. Profitability assessments of the TBLP suggest that a shift of 1% in interest rates has almost identical impact to a shift of 1% in rental yield; i.e. ±£30k and ±£31.2k respectively, over a 10-year period. Since the overall profits from rental income average to just 38.7k, the risk of loss becomes evident when interest rates increase or rental yields decrease by 1.2%, whereas at 1.5% prospective net profits are eliminated since the risk of loss becomes 100%. Naturally, the risk of loss may occur with a lower combined effect associated with these two factors, and this reveals a substantial risk of turning a BTL investment into a lossmaking one (without accounting for any capital gains). Moreover, given that it is reasonable to expect some BTL investments in London to already suffer from these lower rental yields [14] and higher interest rates (refer to Table 5), these BTL profiles are at a significant risk of becoming lossmaking once the new tax reforms come into full effect in tax year 2020/21.

On the other hand, the landlord has control over which managing agent firm to employ, and whether to employ one. Simulation shows that the difference between not employing and employing one of the more expensive letting agencies, for full property management over the 10-year period, has an impact of ±£34.5k on overall net profits (refer to Table 10). Interestingly, the maximum an investor can save from not employing a letting agency is roughly equivalent, as well as limited, to the 1% shifts in interest rate or rental yield. Another control factor is, naturally, the amount borrowed. In fact, reducing LTV provides potential for an investor to indirectly control the interest rate, on the basis that lower LTV rates have access to lower interest rates.

When it comes to capital growth, a shift of 1% can have a highly volatile impact due to its diminishing or cumulative effect on capital gains. For example, for any 10-year period since 1973, where the average capital growth rates hover between 2% and 18%, a shift of 1% on average capital growth results in an impact on gross capital gains ranging anywhere from -£57k to +£229.1k (refer to Table 12).

### Profitability comparison between the old and the new tax reforms

Table 14 provides detailed summary statistics of the return-on-investment (ROI), with and without leverage, with and without selling the portfolio at the end of the 10-year period, and based on both the old and the new tax reforms. Note that, in the case of selling the portfolio, we consider the following additional facts or assumptions:

1. Agent fees from selling the properties are set to 2% (these typically range between 1% and 3%, including VAT).
2. Each property sale assumes a relatively high cost of £5k to cover solicitor and other fees, but which remains constant over the simulation period.
3. The current annual tax allowance from capital gains is £11.1k per individual [61]. This allowance tends to increase marginally per annum. We assume that by the end of the 10-year period the allowance will be £15k. We also assume that the property portfolio has two owners, typically husband and wife, which doubles the tax allowance to £30k per annum. Note that in the case of leverage, we also assume the whole portfolio is sold within a single tax year (i.e. at the end of *t10*) and hence, not benefiting from tax allowances available in other years.
4. Residential tax from capital gains is set to the current rate of 28% [62], which is in line with the *Higher rate* tax band considered by the TBLP (refer to Table 1).

**Table 14:** Overall profit and ROI, based on both the old/current and new tax measures, with and without leverage, and with and without selling the property portfolio. Monetary values represent £GBPs in thousands (k). Note that In the case of leverage, the additional SDLT paid and the additional purchasing costs are already incorporated into *Loan/s*, since these costs are covered by borrowing.

	Prior to the new tax measures		With the new tax measures	
	Without leverage (1 property)	With leverage (8 properties)	Without leverage (1 property)	With leverage (5 properties)
***Portfolio details***				
*Portfolio value*	1130.6k	4304.7k	1130.6k	2428.6k
*Capital*	755.6k	1095.3k	755.6k	1562.4k
*Loan/s*	375k	2742.3k	375k	1333.3k
*Overall LTV*	33.2%	63.7%	33.2%	54.9%
***Initial investment***				
*Deposit*	125k	125k	125k	125k
*SDTL*	30k	30k	30k	30k
*Other purchasing costs*	5k	5k	5k	5k
***Total invested***	**160k**	**160k**	**160k**	**160k**
***Profits if the portfolio is maintained as an investment***				
*Capital gains*	595.6k	1402.4k	595.6k	935.3k
*Net rental profit*	57.1k	103.8k	38.7k	8.69k
***Overall profit***	**652.7k**	**1506.2k**	**634.3k**	**944k**
***ROI***	**408%**	**941%**	**396%**	**590%**
*ROI adjusted for inflation*: *1%*	369.3%	852.2%	358.9%	534.1%
*ROI adjusted for inflation*: *2%*	334.7%	772.3%	325.2%	484%
*ROI adjusted for inflation*: *3%*	303.5%	700.5%	295.0%	439%
*ROI adjusted for inflation*: *4%*	275.6%	636%	267.8%	398.6%
*ROI adjusted for inflation*: *5%*	250.4%	577.9%	243.4%	362.2%
***Additional expenses if the portfolio is sold***				
*Agent fees*	22.6k	86.1k	22.6k	48.6k
*Other selling costs*	5k	40k	5k	25k
*Tax (see Tax allowances)*	142.9k	313.7k	142.9k	212.3k
***Total additional expenses***	**170.5k**	**439.8k**	**170.5k**	**285.9k**
***Tax allowance if the portfolio is sold***				
*Capital gains tax allowance*	30k	30k	30k	30k
*From cost of sales*	27.6k	126.1k	27.6k	73.6k
***Total additional expenses***	**57.6k**	**156.1k**	**57.6k**	**103.6k**
***Profits if the portfolio is sold***				
*Net capital profit*	425.1k	962.7k	425.1k	649.5k
*Net rental profit*	57.1k	103.8k	38.7k	8.69k
*Profit % from capital*	88.16%	90.26%	91.66%	98.68%
*Profit % from rent*	11.84%	9.74%	8.34%	1.32%
***Overall profit***	**482.2k**	**1066.5k**	**463.8k**	**658.2k**
***ROI***	**301%**	**667%**	**290%**	**411%**
*ROI adjusted for inflation*: *1%*	272.8%	603.4%	262.4%	372.4%
*ROI adjusted for inflation*: *2%*	247.2%	546.8%	237.8%	337.4%
*ROI adjusted for inflation*: *3%*	224.2%	496%	215.7%	306.1%
*ROI adjusted for inflation*: *4%*	203.6%	450.3%	195.8%	277.9%
*ROI adjusted for inflation*: *5%*	185.0%	409.2%	178.0%	252.5%

**Fig 8:**
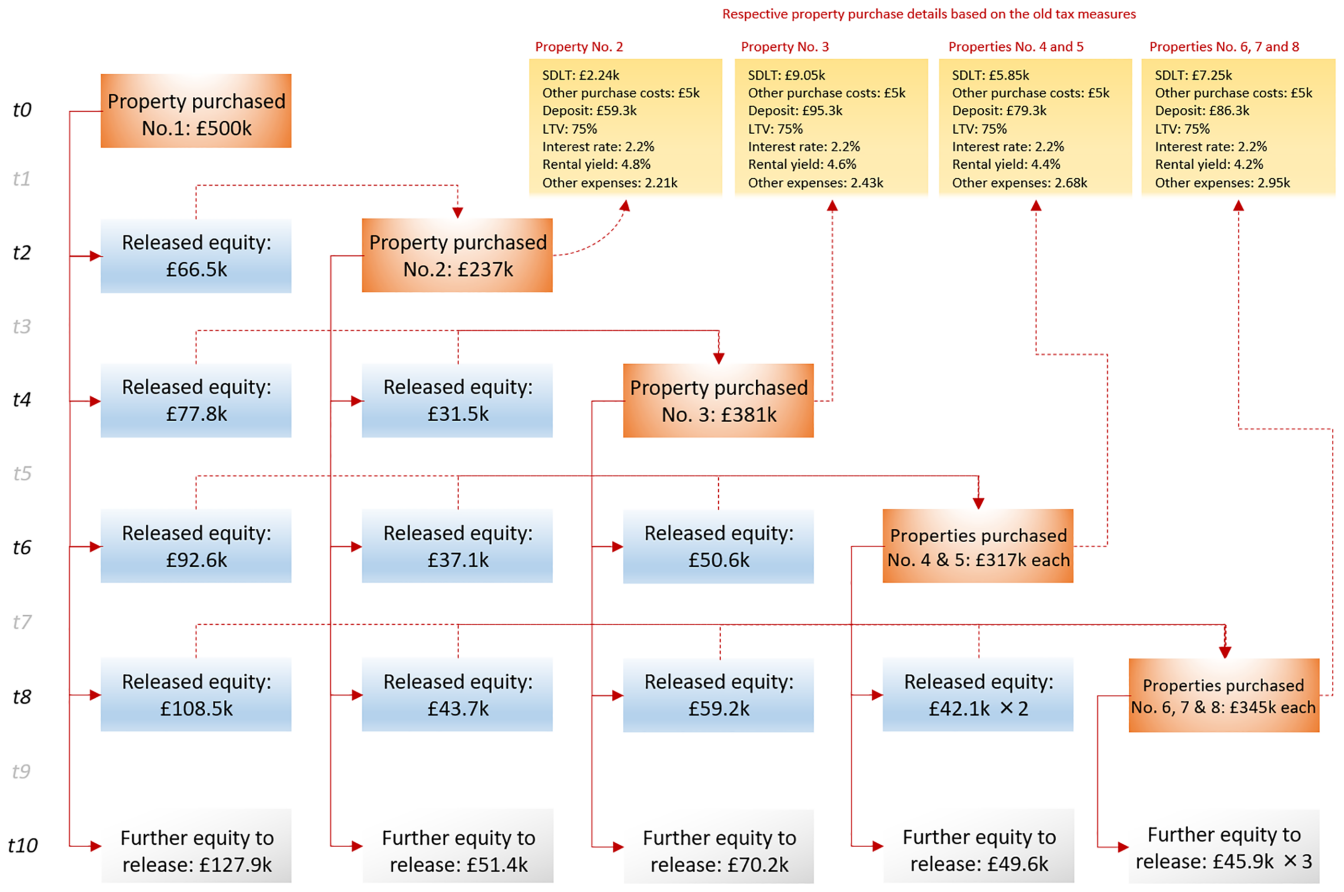
The process of leveraging the TBLP scenario of Fig 6, when based on the old tax measures.

The results show that the expected ROI under the new tax measures ranges from 290% to 591%, depending on whether the investor chooses to leverage and/or sell the portfolio at the end of the 10 years. On the other hand, the expected ROI figures under the old tax measures range from 301% to 944%. The results are distributed between not leveraging and leveraging, and not selling and selling the portfolio at the end of the period. Specifically, the impact on the TBLP is as follows:

1. In the case of *not* leveraging, the new tax reforms decrease ROI from 408% to 396% when the portfolio is maintained as an investment, or from 301% to 290% when the portfolio is sold and capital gains tax is paid. Overall, this represents a rather marginal impact which hovers between 3% and 4%. However, this impact comes exclusively from reducing net profits from rental income, from 57.1k to 38.7k, and which represents a significant impact of -32.2%.
2. In the case of leveraging, the new tax reforms decrease ROI from 941% to 590% when the portfolio is maintained as an investment, and from 667% to 411% when the portfolio is sold and capital gains tax is paid. Overall, this represents a significant impact which hovers between -37% and -38%. Similar to Fig 6, Fig 8 demonstrates the process of leveraging when the figures are based on the old/current tax measures, and demonstrates how the purchase of 8 properties is achieved, up from 5 properties when based on the new tax reforms of Fig 6.

However, given that the expected 10-year ROI under the new tax reforms ranges between 290% and 590% suggests that long-term investment prospects are likely to remain good if current economic circumstances continue. Table 14 also illustrates how ROI adjusts for different inflation rates. According to the Office for National Statistics in the UK [63], the inflation rate for any 10-year period since 1989, and up to and including 2015, hovers between 1.51% and 3.7%. The long-term ROI from BTL investments is projected to be well above these rates.

While leverage is the clear winner when it comes to overall profitability, these profits come almost exclusively in the form of capital gains. As a result, leverage limits cash flow and increases the risk of lossmaking in terms of prospective profits from rental income, and this is a major risk for investors without cash reserves. Additionally, leverage requires substantial more effort by investors, since it involves buying additional properties, obtaining just as many mortgages, as well as dealing with additional demands which inevitably arise from additional tenancies. These are factors an investor needs to assess in order to determine whether the increased risk and effort is worth the projected increase in profitability, under different levels of leveraging.

## Concluding remarks

In 2015 the British government announced a number of tax reforms for individual BTL investors. These new measures increase tax payments for BTL mortgaged investments considerably. To give landlords time to adjust, part of these measures are being introduced gradually from April 2017 with full effect in tax year 2020/21. The paper provides two novel contributions to the state—of-the-art: one is the simulation model itself and the other is the study of a research question which we have not seen before in a real estate context.

The novel TBN model provides the capability to an investor to simulate the impact of various factors and interventions of interest on a BTL investment, and over a 10-year period. The model captures uncertainties of interest and permits for intervention between time steps to allow for risk management of changing circumstances, such as changes in interest rates and rental and capital growths. The temporal Bayesian modelling technique appears to be well suited to address the research question. Unlike past relevant publications which tend to focus on analysing and predicting housing market trends, this paper assesses the prospective performance of BTL investments from the investor’s perspective, and examines the impact of incoming tax reforms. The analysis focuses on the London BTL property market and assumes a typical BTL London profile, which we call TBLP, in assessing the impact of various factors of interest, in conjunction with tax reforms. The main conclusions from simulation are:

1. The new tax reforms are projected to have a significant impact on net profits from rental income. Overall, the new tax measures demonstrate high risk in terms of eliminating profits and transforming a BTL investment into a lossmaking investment (excluding capital gains). However, it is important to note that this outcome does not imply that future BTL investors are expected to generate such losses, simply because lenders are likely to take (and already are) a more conservative approach to their rental stress testing and affordability calculations to ensure that borrowers will be in position to meet mortgage payments (i.e. by requesting larger deposits to ensure that rental income will be sufficient to cover mortgage payments and other costs associated with the investment).
2. Property prices in London have been increasing faster than rents. If this trend continues, simulation suggests that rental yields in London will continue to decrease by 0.1% per annum. In fact, Portico [14] show that in 2012 half of London was generating rental yields in excess of 6%, whereas since 2015 all London districts generate rental yields below 6%. In reality, this trend cannot continue for much longer. Either capital growth rates will have to decrease, rental growth rates will have to increase, or we shall observe a combination of the two prospective events.
3. Historic low interest rates add substantial uncertainty to BTL investments. It is not clear whether we have entered an era of extremely low interest rates, or whether the Bank of England has plans to increase them. If interest rates rise by an absolute 1.5% under the new tax reforms, this poses a major risk of collapse of the mortgaged BTL market. However, interest rates are unlikely to increase by this much unless there is strong economic activity, which should in turn have a positive effect on the growth of both the rental income and capital gains. Further, interest rates are controlled by the Bank of England, and it should be in their best interest not to cause chaos in the UK property market.
4. The significant impact on net profits from rental income poses considerable risk to investors with no cash reserves. This makes the prospect of investors intervening on controlled costs more likely, such as reducing costs related to managing agencies, or limiting leverage to achieve better interest rates through decreased LTV rates.
5. The new tax reforms hit risk-seeking BTL investors who favour leverage much harder than risk-averse investors who do not expand their property portfolio. If lenders introduce stricter criteria for borrowers in light of the tax reforms, the investors’ ability to leverage will diminish further. The projected limitation on leveraging will also have a negative effect on demand for property. However, the level of impact on demand is unclear. We have no information with respect to what proportion of property demand is attributed to leveraging. Given that property demand in London is mainly driven by the growing population and the limited housing supply [64], it is unlikely these events will have major impact on overall demand for property.
6. Since the ROI from BTL investments is predominantly driven by capital growth, profitability prospects remain good under the new tax reforms, even when ROI is adjusted for high inflationary rates. In an era of negative prospects about economic stability, and in conjunction with the uncertainties surrounding Brexit in the UK, it may be difficult to foresee how these capital growth rates will be repeated. However, one could argue that such disbeliefs are never-ending and have been refuted multiple times in the past. Regardless, and further to what has been discussed in point (5) above, it is reasonable to expect the capital growth rates to decrease in the near future. Our analyses do not make it possible for us to comment on how capital growth rates are expected to change. However, what we do know is that If we base capital growth expectations on the worst 10-year period observed since 1973, which translates to an average of a 2% annual capital growth (refer to Section 6.5), and with all the other factors unchanged, the most adverse expected ROI figure over the 10-year period (i.e. without leveraging and after paying capital gains from selling the portfolio) is 69%. Also, according to official Land Registry statistics, the annual capital growth in June 2016 (i.e. pre-Brexit referendum) was 13.82% for London and 8.01% for the UK, whereas in March 2017 the respective rates are down to 1.53% and 4.1%, and continue to follow a decreasing trend. Part of this decrease can also be attributed to the SDLT changes (refer to Table 3), as well as to the incoming BTL tax reforms. The excessive capital growth observed post-2009 crisis can also be seen as a cause of the decreasing growth trend, simply because it is natural to observe a slowdown following excessive growth (and vice versa). The overall complexity of the situation makes it difficult to establish the real effects of the various political events and policy tax interventions on the housing market.

Our aim was to assess the impact of the new tax reforms, and in doing so we considered a common London property profile. London itself, however, consists of boroughs with major differences in terms of property value and demand. For example, the most extreme case between boroughs in 2016 (up to August), shows that property sales in the London borough of *Kensington and Chelsea* averaged £1.91m, which is 678% higher than the average sale of £282k in the borough of *Barking and Dagenham*. The results from simulation (refer to Section 6) allow readers to examine how various changes of the key profile inputs influence the BTL investment, and give an indication as to how profitability might change under different circumstances, events, and BTL profiles.

Overall, while the risk of making a loss from rental income is substantial under the new tax measures, and which makes it less desirable or even non-viable for some to continue being a landlord, investment prospects are likely to remain good within a reasonable range of interest rate and capital growth rate variations. Given that the results are based on a typical BTL profile in London, this implies typical rental yields, expenses, and growth rates. Such a profile, however, underestimates the ability of savvy investors in selecting a BTL property for investment purposes. This is because a savvy investor is expected to scrutinise the relevant performing factors, such as rental yield and capital growth, and make calculating decisions which promise greater profitability. On this basis, the prospective profitability reported in this paper is expected to be greater for savvy investors.
